# Natural antioxidants for health promotion and disease prevention

**DOI:** 10.3389/fphar.2014.00266

**Published:** 2014-12-02

**Authors:** Xing Zhang, Feng Gao

**Affiliations:** Insulin Group, Department of Physiology, School of Basic Medical Sciences, The Fourth Military Medical UniversityXi'an, China

**Keywords:** natural antioxidants, reactive oxygen species, herbs, spices, chronic disease

All biological systems functioning in aerobic conditions are exposed to reactive oxygen species (ROS), a class of oxygen metabolites that are highly active in terms of oxidative modification of proteins, lipids, and DNA. ROS has been found to contribute to aging and quite a number of chronic diseases including diabetes, cardiovascular diseases, neurological disorders, and cancer. Thus, reducing ROS has become one of the most promising approaches to health promotion, disease prevention and intervention of chronic diseases (Firuzi et al., 2011). Compared with synthetic antioxidants, natural antioxidants from plants are considered to be more acceptable, reliable, and safer, which caters for a growing nature-conscious public in quest for natural remedies in promoting health and preventing disease.

The author Denys J. Charles, director of Research at Frontier Natural Products Co-op, has covered in this book almost all of the latest progresses in this field, presenting biological and medicinal aspect of herbs, spices and other sources with the analysis of functional constituents, and provided abundant information of natural antioxidants from these sources. Plants contain high concentrations of numerous antioxidants and these antioxidants have potential therapeutic effects to chronic diseases (Munir et al., 2013). This not only meets the growing demand of the public for daily health and disease prevention, but also offers a comprehensive and up-to-date review for researchers in healthcare and related fields. Dr. Charles starts by describing methods in assessment of antioxidant capacity (30 pages). This is an important issue since there is no standard method in measuring antioxidant capacity of antioxidants and results of antioxidant capacity measured with different assays are incomparable. Then the author delineates properties of various natural antioxidants and their different sources (56 pages), in which plenty of interesting and thought-provoking information are given. For example, the waste of some foods, including peels, seeds, and stems, usually contains higher level of antioxidants, which provides a huge opportunity for industry to obtain ingredients for functional food or medicine. At last, the individual herbs and spices are described in the second part of the book in details, including descriptions of their botany, constituents, and functional properties, as well as some readable information such as history, flavor, aroma, consumption, and medical uses (447 pages). As the most important chapters of the book, this part covers 52 herbs and spices which are commonly used.

It has been a long history of using herbs and spices for human to extend and improve quality of life. These herbs and spices have low calorie content and contain high levels of antioxidants and other potential bioactive compounds. Thus, it is essential to create awareness in society about the reliability of medicinal properties of certain herbs and spices through scientific research with analysis of constituents, toxicology, and physiological effects. In this regard, the book gives the reader comprehensive information about the diversity of the natural antioxidants abundant in herbs and spices that may contribute to the beneficial effects of these plants in health promotion and disease prevention. One of the most impressive features about this book is that in terms scholarship, the author has combed with care published studies in this field, which is characterized by tremendous well marked references from well established “main stream” journals, which facilitates further research in this area.

However, this book uncovered several important issues for scholars. For example, it is not clearly addressed to what extent these natural antioxidants can be absorbed by target organs or tissues and to what extent the antioxidant capacity of these natural antioxidants retains *in vivo*. More importantly, it is still unknown how much these natural antioxidants can contribute to the total beneficial effects of these plants in health promotion and disease prevention and how much these natural antioxidants exert beneficial effects through directly reducing oxidative damage rather than other biological functions (Madeo et al., 2014), since most clinical trials of natural antioxidants in the treatment of various diseases produced negative results. Finally, some illustrating pictures would make this book more readable.

Despite the limitations, this book provides more detailed information than is available anywhere else for researchers in antioxidant study from both academia and industry, for the health professionals who are looking to give their patients with chronic diseases adjunctive therapies, and for the general public in their smart choice of natural antioxidants to improve their health.

## Conflict of interest statement

The authors declare that the research was conducted in the absence of any commercial or financial relationships that could be construed as a potential conflict of interest.
